# Monomicrobial Staphylococcus aureus Necrotizing Soft Tissue Infection: A Case Report and Review of Literature

**DOI:** 10.7759/cureus.115915

**Published:** 2026-09-07

**Authors:** Ryan Vandenbord, Emma Amjad, Hadi F Shaaban

**Affiliations:** 1 Medicine, Edward Via College of Osteopathic Medicine, Auburn, USA; 2 Surgery, HCA Florida Fort Walton-Destin Hospital, Ft Walton Beach, USA

**Keywords:** debridement, early debridement, fascitis, lrinec, methicillin resistant staphylococcus aureus (mrsa), necrotizing soft tissue infection (nsti)

## Abstract

Necrotizing soft tissue infections (NSTIs) are severe infections that cause destruction of tissues, including the skin, fascia, and muscle. Here, we discuss a case of a monomicrobial NSTI caused by methicillin-resistant *Staphylococcus aureus* (MRSA). The patient presented with signs of sepsis and a large, pustular, purulent, and draining skin infection, consisting of 50-60 lesions that had coalesced into a large collection in the right lumbar region and tracked inferolaterally toward the buttocks. This case provides an opportunity to discuss the pathophysiology underlying the progression of MRSA abscesses to NSTIs, as well as the diagnosis and treatment of these infections. The patient was treated with intravenous antibiotics and required surgical debridement, which revealed extension of the infection into the subcutaneous tissue, muscle, deep lumbar fascia, and posterior layer of the thoracolumbar fascia. This case highlights the unusual anatomical location and monomicrobial etiology of an NSTI. It also underscores the diagnostic challenges in determining the severity of infection, as existing diagnostic tools for NSTIs may have limited utility in atypical anatomical regions and in infections arising from uncommon sources.

## Introduction

Necrotizing soft tissue infections (NSTIs) are severe, life-threatening infections characterized by rapid destruction of soft tissue such as the skin, fascia, or muscle. In the United States, NSTIs have an incidence of 3.8 to 10.3 cases per 100,000 persons annually [1]. The peak incidence of NSTI is reported in Thailand, where it reaches 15.5 per 100,000 persons annually. Worldwide, the incidence ranges from 0.2 to 6.9 cases per 100,000 individuals annually [2]. These infections are categorized into two main categories: type 1 (polymicrobial) and type 2 (monomicrobial). Type 1 NSTIs are more common, responsible for 53% of cases seen globally, whereas type 2 NSTIs are responsible for 38% of cases. The remaining 9% of cases consist of type 3 infections, with etiologic agents being either *Vibrio vulnificus* or *Aeromonas hydrophila*, and type 4 infections, which are fungal in origin. Type 2 NSTIs are most commonly due to Group A *Streptococcus* infections, while the second most common pathogen is methicillin-resistant *Staphylococcus aureus *(MRSA)* *[2]. These infections most commonly affect the extremities (57-73%) and less commonly affect the trunk (13-26%) [1].

Here, we discuss the case of an NSTI affecting the trunk in an otherwise healthy middle-aged male patient with no comorbid conditions. This individual’s history of a three-week rash displayed the progression from cellulitis, to multiple abscesses, and subsequent NSTI. This article aims to review the pathogenesis and presentation of NSTIs and commonly used diagnostic tools. NSTIs are often identified based on common physical presenting signs, namely bullous, violaceous skin lesions. The clinical presentation of this patient’s NSTI did not follow these classic characteristics, which is suspected to be due to the causative organism. This case is unique in terms of the etiologic organism, physical appearance, and physical location. Identification and documentation of an NSTI with a unique clinical presentation, such as this patient’s, is imperative to add to the understanding of how NSTIs due to lesser common pathogens present. Early identification of the severity of this infection was imperative in treating the subsequent complications of infection, namely sepsis, and operative intervention was crucial in preventing progression to septic shock. 

## Case presentation

A 55-year-old male patient presented to the emergency room with a chief complaint of a large wound on his lower back associated with pain, redness, and purulent discharge. The patient stated that he first noticed the rash three weeks ago, which was originally present on his right thigh. He described the rash as blistering, sore, and itchy. Over this three-week time frame, the rash had progressed to involve his right lower back. The patient stated that the rash was itchy and that he had been scratching the area to alleviate discomfort. He originally expressed that he was concerned the rash originated from a shingles infection, which he believed began three weeks ago when the rash first appeared. The patient did not seek any medical care or receive a formal diagnosis to confirm this suspicion. The patient denied prior vaccination history against shingles. Over the prior two days, he noticed increased yellow drainage from the area, associated with increased pain and redness. He stated that he did not feel febrile.

The patient also reported a history of cellulitis of the second toe of the right foot six months prior. He stated that the pain had been persisting for the last six months and thought that it was due to a fishbone stuck in his toe. He was treated with cephalexin 500 mg capsule four times daily for seven days and reported no further concerns. He also stated that he did not see his primary care provider regularly and had no medical history. He denied a history of intravenous drug use, diabetes, or immunosuppression. Despite his previous suspicion, the patient denied a history of shingles.

Upon presentation to the emergency room, the patient’s vital signs included a temperature of 101.7 degrees Fahrenheit, a heart rate of 91 beats per minute, a respiratory rate of 14 breaths per minute, and a blood pressure of 108/70 mmHg. The patient’s labs were significant for a white blood cell count of 20.93 × 10^3^/µL (Table 1). 

**Table 1:** Laboratory values for the day of presentation through day three after surgery and the day of discharge POD: postoperative day, WBC: white blood cell, RBC: red blood cell, Hb: hemoglobin, Hct: hematocrit, CO2: Carbon dioxide, BUN: blood urea nitrogen, CBC: complete blood count, BMP: Basic metabolic panel. This data has been extrapolated to diagnose the patient with sepsis at the time of his arrival using Systemic Inflammatory Response Syndrome (SIRS) criteria. The data was used to calculate the patient's Laboratory Risk Indicator for Necrotizing Fasciitis (LRINEC) score.

Labs ordered	Admit	Preoperative day 1	POD 0	POD 1	POD 2	POD 3	POD 10	Reference Range
Complete Blood Count with Differential								
WBC	20.93	20.99	16.37	14.11	14.95	10.61	8.65	4.23-9.07 x 10^3^/mL
RBC	4.20	4.02	4.13	3.86	3.27	3.39	3.10	4.63-9.07 x 10^3^/mL
Hemoglobin	12.1	11.4	11.7	10.9	9.5	9.5	8.8	13.7-17.5 g/dL
Hematocrit	35.7	35.4	36.0	33.7	28.8	29.4	27.7	40.1-51.0%
Platelet count	364	421	415	520	478	536	444	163-337 x 10^3^/mL
Absolute Neutrophils	17.76	17.70	13.74	11.67	7.10	6.26	5.75	(1.78-5.38 x 10^3^/mL)
Absolute Lymphocyte	1.01	1.10	1.06	.97	1.82	1.46	1.65	(1.32-3.75 x 10^3^/mL
Basic Metabolic Panel								
Sodium	136	138	139	140	143	141	143	136-145 mmol/L
Potassium	3.3	3.9	4.2	4.4	4.0	4.2	3.7	3.4-5.1 mmol/L
Chloride	96	100	103	103	104	104	106	98/107 mmol/L
BUN	8	5	<5	12	8	13	14	9-23 mg/dL
Creatinine	0.65	0.69	0.65	0.71	0.68	0.67	0.63	0.7-1.30 mg/dL
Calcium	9.3	8.7	9.2	8.7	8.4	8.6	8.1	8.7-10.4 mg/dL
Glucose	104						90	46-116 units/L
Serology								
HIV 1&2 Ab/P24 Ag fourth-generation			Nonreactive					

**Figure 1:**
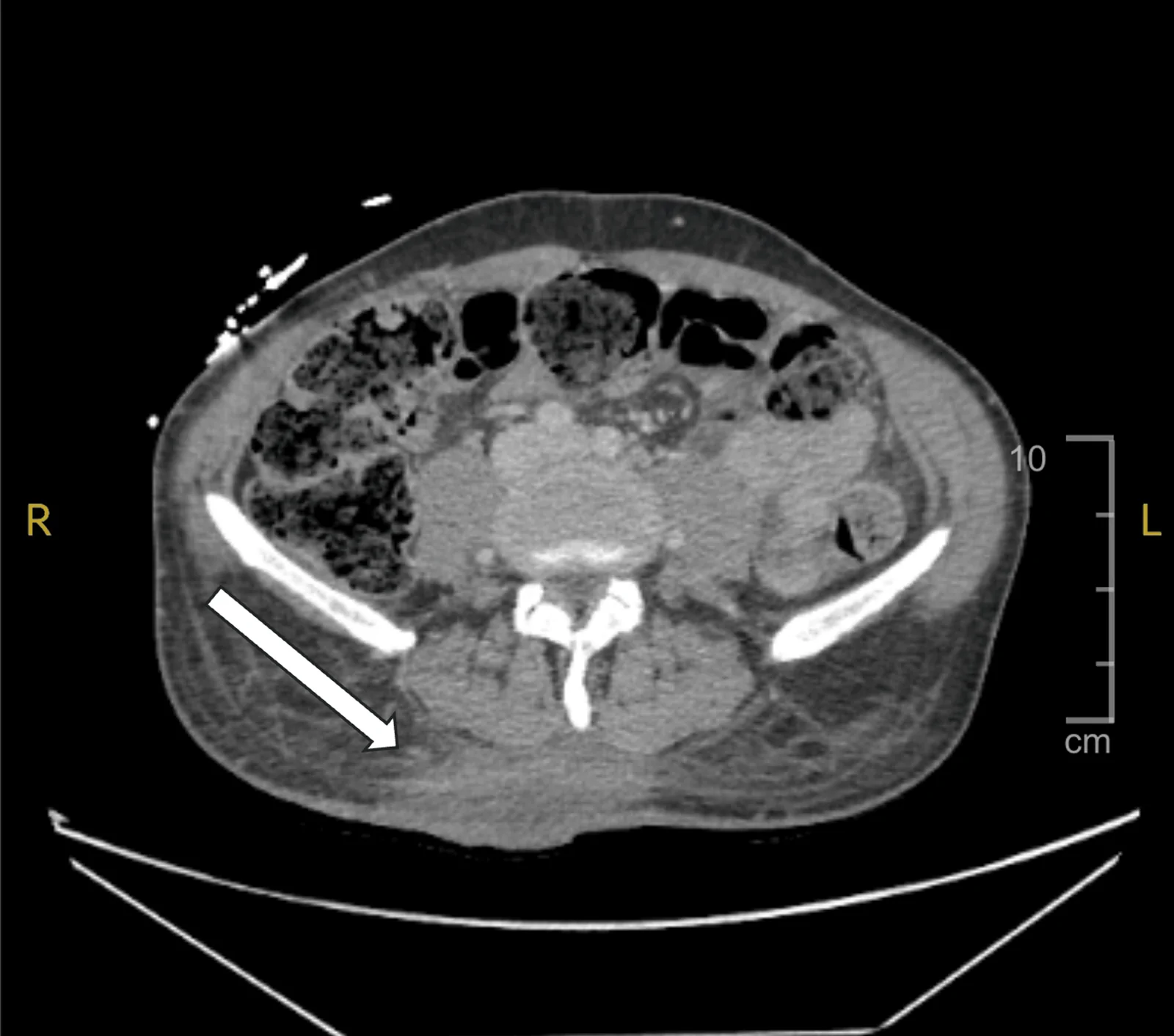
Computed tomography (CT) axial view of the abdomen and pelvis The image displays subcutaneous 5.8 x 4.6 x 10.3 cm multiloculated abscess in the right mid-to-lower lumbar subcutaneous tissue with surrounding fat stranding and skin thickening, poorly delineated from adjacent muscle fascia and without connection to the skin surface (arrow).

The patient met three out of four systemic inflammatory response syndrome (SIRS) criteria, given his fever was greater than 100.4 degrees Fahrenheit, heart rate greater than 90 and white blood cell count greater than 12,000/mm^3^. A computed tomography (CT) of the abdomen and pelvis was ordered which revealed a subcutaneous 5.8 x 4.6 x 10.3 cm multiloculated abscess in the right mid-to-lower lumbar subcutaneous tissue with surrounding fat stranding and skin thickening, poorly delineated from adjacent muscle fascia and without connection to the skin surface (Figure 1).

Sepsis protocol was initiated and the patient was admitted to medicine service for management of sepsis and hypokalemia. He was started on empiric antibiotics with clindamycin 900 mg, 1000 mg vancomycin, and 1000 mg ceftriaxone in the emergency room. During his hospital course, he was continued on vancomycin 1000 mg every eight hours. The patient was resuscitated with a 2500 cc bolus of normal saline and placed on 75 cc/hr maintenance fluids. A culture of the patient’s wound was taken, and an automated microbial detection system revealed a monomicrobial *Staphylococcus aureus* infection. Blood cultures were also drawn, which were negative for any organisms.

The Infectious disease department was consulted. It was recommended that the patient be continued on ceftriaxone and vancomycin therapy. Given the patient’s previous mention of a shingles infection, this causative source was clinically ruled out. Since the patient had not been diagnosed with any previous medical conditions, he was tested for HIV to identify any possible immunosuppression leading to his infection. A HIV 1&2 Ab/P24 Ag fourth-generation test was negative, ruling out HIV (Table 1).

The surgical team discussed the indications for surgery in patients with NSTIs and informed him of the severity of his condition. However, the patient opted to proceed with non-operative treatment. After a lack of improvement in his symptoms in the next 24 hours, the patient was counseled again on the severity of his condition, and the decision to proceed with surgery was made.

The patient was taken to the operating room and placed in the prone position. Electrocautery was used to excise the skin and subcutaneous tissue starting in the center of the abscess most proximal to the center of the affected area. The excision was performed circumferentially, extending deep through the subcutaneous tissue, the first layer of muscle, and the fascia until the posterior lumbar fascia was identified. During the procedure, it was noted that the necrotizing tissue had spread through these layers. Excision was therefore extended beyond the affected area to the first discernible plane of unaffected tissue, which was the posterior lumbar fascia. Excision of this tissue was performed over an area measuring 17 × 19 cm, with additional areas measuring 12 × 10 cm and 5 × 5 cm also excised in a similar fashion. Once the necrotizing tissue was removed, the area was dressed with Silvadene slurry, kerlix rolls, 4 x 4s and abdominal pads. The patient was then transferred into the post-surgical care unit without complication.

The patient’s dressing was removed on the postoperative day (POD) one and replaced with a wet to dry dressing. A wound vacuum was placed on POD three. Pathologic evaluation of the surgical specimen, using a automated microbial detection system, confirmed a monomicrobial MRSA infection. Culture sensitivity results revealed vancomycin sensitivity. The patient stayed for an additional 10 days in the hospital for wound care and continued intravenous antibiotic therapy. Over the duration of his stay, the patient’s WBC count started at 20.93, was 14.11 on POD one, and was 8.65 at discharge (Table 1). He was discharged to a skilled nursing facility for further management of his wound vacuum and intravenous antibiotics.

## Discussion

NSTIs are life-threatening infections that affect the skin, subcutaneous tissue, muscle, and fascia and can progress to multisystem organ failure in later stages of disease. Four types of these infections exist: Type 1 (polymicrobial infections), Type 2 (monomicrobial infections), Type 3 (infections due to *Vibrio vulnificus* or *Aeromonas hydrophila)*, and Type 4 (fungal infections). This case represents a rare monomicrobial infection, which makes up only 38% of infections worldwide, with *S. aureus* being the second most common causative organism [1].

Abscess formation due to *S. aureus* occurs in four main steps: initial insult and invasion, innate immune activation and neutrophil recruitment, pseudocapsule formation, and maturation and immune evasion [3]. *S. aureus* penetrates the skin through a break in the skin barrier or mucosal integrity, such as skin trauma or incision sites. Microbial surface components recognizing adhesive matrix molecules (MSCRAMM) mediate adhesion to the host tissue by binding to fibronectin, fibrinogen, von Willebrand factor, or collagen [4]. Innate immunity is evaded through a variety of mechanisms, including Panton-Valentine Leukocidin (PVL), which impairs innate immunity via lysis of neutrophils by binding C5a receptors [5]. *S. aureus* contains cell wall components such as lipoproteins (LPP) and peptidoglycan (PG), which work together to activate Toll-like receptor 2 (TLR2) and Nucleotide-binding Oligomerization Domain-containing protein 2 (NOD2), which increase levels of Macrophage Inflammatory Protein (MIP-2) aka CXCL2, and facilitate further abscess formation. MIP-2/CXCL2 increases neutrophil activation and attraction, contributing to abscess proliferation [4,6,7]. The third step involves the formation of structured abscess communities and the formation of two coagulase-dependent barriers. These barriers consist of an inner fibrin pseudocapsule formed by activation of prothrombin by coagulase and an outer microcolony-associated meshwork [4,7]. Finally, a mature abscess is formed, which consists of a central bacterial colony surrounded by the pseudocapsule formed as a mechanical barrier to immune evasion.

The transformation from a contained abscess to an NSTI occurs through a variety of mechanisms. The primary mechanism of endothelial injury and dermonecrosis in NSTI caused by *S. aureus* is mediated by alpha-hemolysin, which induces endothelial cell apoptosis via binding to A Disintegrin And Metalloproteinase domain-containing protein 10 (ADAM10) on host cells, leading to cleavage of vascular endothelial (VE)-cadherin. As a result, vascular injury occurs, leading to hypoxia and subsequent keratinocyte apoptosis. In addition, alpha hemolysin activates the NLR family pyrin domain containing 3 (NLRP3) inflammasomes in monocytes, leading to caspase-1 activation and subsequent release of High Mobility Group Box (HMGB1), IL-1beta, and IL-18. These inflammatory mediators and cytokines act to further amplify the inflammatory cascade, although keratinocyte apoptosis triggered by ischemia is the primary mediator of necrosis [3,8]. Through these mechanisms, the toxin-mediated inflammation causes endarteritis and thrombosis, leading to tissue ischemia and necrosis. Aggregates of platelets and leukocytes occlude capillaries leading to thrombosis of fascial vessels, precipitating a cascade of events. Progressive vascular occlusion causes perforation of skin vessels and secondary ischemia and tissue hypoperfusion, which creates an anaerobic environment for other bacterial growth, impairing delivery of antibiotics to tissues [1,2].

Once an NSTI is suspected, it is largely a clinical diagnosis, but multiple clinical tools exist to assist in diagnosis. Many early warning signs exist, such as erythema, pain, swelling, while more specific findings such as bullae and gas on X-ray may prove more diagnostic [9]. CT scan findings have been shown to have an 89% sensitivity and 93% specificity for detecting an NSTI, indicating its value as a diagnostic tool in patients with an indeterminate probability of having it [10].

The laboratory risk indicator for necrotizing fasciitis (LRINEC) score is used to evaluate the probability of necrotizing fasciitis and has shown mixed results in accurately diagnosing it in multiple large retrospective studies. This scoring system is associated with a positive predictive value of 92% and negative predictive value of 96% to differentiate necrotizing fasciitis from cellulitis. When using a score of six as the cutoff for diagnosis, sensitivity and specificity ranged from 49-75% and 83-85% respectively. When a score of eight was used as the cutoff for diagnosis, sensitivity and specificity ranged from 41% and 95% respectively [10,11]. Another clinical reasoning tool is necrotizing soft tissue infections scale (NECROSIS), which evaluates patients on three independent predictors: systolic blood pressure of 120 mmHg or less, violaceous skin, and white blood cell count over 15,000/uL. This analysis showed that if these three factors are present, there was a 100% sensitivity and positive predictive value in diagnosing NSTI in their subgroup analysis [12].

Given the difficulties in diagnosing NSTIs, operative exploration may be necessary when a clear clinical diagnosis is not made. Operative findings of NSTI include dishwater appearance of fluid, gray appearance of tissues, necrotic fascia, obliteration of soft tissue planes, and lack of bleeding [1]. In many cases, one operation is not enough to both diagnose and treat the infection fully, with re-exploration every 12-24 hours being indicated after the initial operation of source control and when worsening signs of infection are present [13].

Overall, the treatment of suspected NSTI includes broad spectrum antibiotic therapy, which should be initiated after obtaining blood cultures. Antibiotic coverage includes coverage for gram-positive, gram-negative, and anaerobic organisms. Vancomycin 25-30 mg/kg loading dose, then 15-20 mg/kg every eight hours or linezolid 600 mg every 12 hours should be used in suspected MRSA infections. Clindamycin 600-900 mg every eight hours should be added in patients that are unstable with systemic symptoms that may indicate toxic shock syndrome [1].

Treatment of NSTI with surgical debridement is crucial in preventing progression to septic shock. Kobayashi et al. compared outcomes of early (<12 hours) and late (>12 hours) debridement in the treatment of NSTI. This review showed that early intervention was associated with improved mortality, 14% vs 26% respectively [14]. In the case at hand, an attempt was made within this early window to treat the patient with surgical intervention, but the patient opted to proceed with antibiotics rather than surgery. It is important to note that the patient did not meet the criteria for a high or even moderate probability of NSTI using the LRINEC score and was stabilized after fluid resuscitation in the emergency room. Given this fact, the timing of his surgery should be viewed within the greater context of his presenting symptoms and shared decision making with the patient.

Overall, this case presents the difficulties and nuances involved in making a diagnosis of an NSTI, especially given the monomicrobial nature of the patient’s infection leading to atypical presentation of NSTI. Based on the pustular appearance of the infection, it was originally suspected, on admission to the emergency room, that the patient was suffering from a complex, multiloculated abscess. This clinical presentation provided a challenge in diagnosis. Given this complex clinical picture and presence of lateral tracking, CT was a valuable tool to evaluate the extent of the infection. It identified a multiloculated abscess with fat stranding, skin thickening, and poorly delineated adjacent muscle fascia. The LRINEC score of the patient discussed in this review was two, indicating a low risk for necrotizing fasciitis, although this score is not reliable being that a CRP was not ordered. This poses a diagnostic challenge as CRP is not necessarily a standard laboratory test to order for every patient with NSTI but may prove a valuable adjunctive test especially if the LRINEC score is used as a regular diagnostic tool. The NECROSIS scale is another clinical diagnostic tool but may not prove to be useful in type II infections, especially in less common monomicrobial etiologies such as MRSA. Our patient did not fully meet the criteria based on this guideline, as a violaceous appearance was not present.

Although these tools have proven reliable in helping increase the index of suspicion in diagnosing NSTI, operative exploration is necessary in many cases, as it was in this one. Once the patient was taken to the operating room and surgical excision took place, necrotizing tissue affecting tissue planes superficial only to the posterior lumbar fascia was noted. These findings are pathognomonic for NSTI, and are consistent with operative findings listed in a review by McDermott et al. which included lack of bleeding, thrombosed vessels, lack of resistance to finger dissection, noncontracting muscle, and obliteration of soft tissue planes [1]. Ultimately, our patient was treated with vancomycin 1000 mg, 1000 mg ceftriaxone and clindamycin 900 mg originally, and therapy was de-escalated to vancomycin 1000 mg. This antibiotic therapy is consistent with the standard of care. Treatment with operative intervention was crucial in preventing the progression of his condition into septic shock.

Ultimately, this case presents a vital overview of an NSTI in an uncommon location due to an uncommon organism. As discussed previously, multiple diagnostic tools exist to aid in diagnosis of NSTI, with surgical exploration and debridement being a definitive measure for diagnosis and treatment. 

## Conclusions

NSTIs are serious, life-threatening infections affecting the skin, soft tissues, muscles, and fascia, most commonly in the extremities and less commonly in the trunk. Here, we present a patient with an NSTI with a three-week history of a purulent draining abscess presenting to the emergency room with signs of sepsis, which was subsequently treated with intravenous antibiotics and surgical excision. This case provides an example of a rare monomicrobial etiology of NSTI, namely *S. aureus, *that did not present with classic signs of necrotizing infections, such as purple hue and bullae. Given this atypical visual presentation, the definitive diagnosis came later in the operating room. This is common in NSTI diagnosis and treatment. Clinical diagnostic tools in this case were not useful adjuncts for diagnosis, which may have been due to the less common etiology of this type 2 infection.
